# MD-CTS: An integrated terminology reference of clinical and translational medicine

**DOI:** 10.1016/j.csbj.2016.02.004

**Published:** 2016-03-02

**Authors:** Will Ray, Joe Finamore, Majid Rastegar-Mojarad, Chris Kadolph, Zhan Ye, Jacquie Bohne, Yin Xu, Dan Burish, Joshua Sondelski, Melissa Easker, Brian Finnegan, Barbara Bartkowiak, Catherine Arnott Smith, Umberto Tachinardi, Eneida A. Mendonca, Bryan Weichelt, Simon M. Lin

**Affiliations:** a Biomedical Informatics Research Center, Marshfield Clinic Research Foundation, Marshfield, WI, USA; b School of Medicine and Public Health, University of Wisconsin, Madison, WI, USA; c Marshfield Clinic, Marshfield, WI, USA; d George E. Magnin Medical Library, Marshfield Clinic, Marshfield, WI, USA; e School of Library and Information Studies, University of Wisconsin, Madison, WI, USA; f National Farm Medicine Center, Marshfield Clinic Research Foundation, Marshfield, WI, USA

**Keywords:** Database, Software, Medicine, Dictionary

## Abstract

New vocabularies are rapidly evolving in the literature relative to the practice of clinical medicine and translational research. To provide integrated access to new terms, we developed a mobile and desktop online reference—Marshfield Dictionary of Clinical and Translational Science (MD-CTS). It is the first public resource that comprehensively integrates Wiktionary (word definition), BioPortal (ontology), Wiki (image reference), and Medline abstract (word usage) information. MD-CTS is accessible at http://spellchecker.mfldclin.edu/. The website provides a broadened capacity for the wider clinical and translational science community to keep pace with newly emerging scientific vocabulary. An initial evaluation using 63 randomly selected biomedical words suggests that online references generally provided better coverage (73%-95%) than paper-based dictionaries (57–71%).

## 1 Introduction

When a clinical or translational scientist encounters a new term, Google is among the top resources utilized to find a definition because 1) it is generally available and easy to use, 2) it is free of charge, and 3) better tools are lacking. While dictionaries represent an alternative resource, paper-based dictionaries are not updated frequently, and therefore do not contain new terms. The desirable tool should have near real-time updates as new terms appear in scientific literature. In addition, the tool should illustrate the usage of the term in a scientific context. Besides textual formats of usage, images surrounding the text can also be helpful. An image can often augment a textual definition, serving as an aid to learning and advancement of comprehension. Currently, no online resource implements and integrates all of the above functionalities.

To address this gap and establish an updated and comprehensive collection of terms used in the clinical and translational medicine domain, we created the Marshfield Dictionary of Clinical and Translational Science (MD-CTS). We utilized an automated approach and adopted the philosophy of Zeng et al. [1] to construct a dictionary by observing the “actual utterances” of scientists and clinicians in scholarly communications. As a proof of concept, we chose Medline abstracts as the source. Our tool is complementary to the search engine found at the PubMed portal providing free access to Medline [2]. Although PubMed permits search of 24 million Medline records, its main functionality is as a query tool and not as a tool for defining terms.

Although usage examples of a term can be obtained automatically by computer, accurate definition of the term has to go through a human editorial process. In contrast to the editorial management by companies who create paper-based dictionaries, Wiktionary crowdsources the editorial effort to the general public via the Internet [3]. As such, it can be updated relatively rapidly. Thus, Wiktionary was integrated into our query website as a look-up tool for those seeking definitions of newly emerging terminology.

Besides verbal definition, ontology can further elaborate the relationship between terms. To provide access to a library of biomedical ontologies and terminologies, BioPortal [4] was developed by the National Center for Biomedical Ontology (NCBO). Currently, BioPortal covers 372 ontologies including Unified Medical Language System (UMLS) and the Medical Subject Headings (MeSH), Systematized Nomenclature of Medicine (SNOMED), International Classification of Diseases (ICD-9), and Gene Ontology (GO). MD-CTS includes BioPortal search results to provide users with the ontological context of a term.

In this paper, we describe how the MD-CTS tool integrates several available resources to bring together concept definition, ontological definition, example usage in context, related terms, and related images from the rapidly expanding repository of clinical and translational medical terminology. The effectiveness of MD-CTS was comprehensively evaluated against traditional dictionaries and other online resources.

## 2 Methods

Words in MD-CTS were extracted from Medline abstracts on a quarterly basis. The first download and parsing in Q4 2013 consisted of 24,557,663 xml files in a relational database of terms in the abstracts and titles. For each word, the database evaluated the frequency of appearance and positional information. A list of 2,486,591,581 words was created via splitting the text with spaces created. Some pre-processing steps such as removing stop words, punctuation, and numbers, resulted in a list of 1,795,769 unique words. MD-CTS's server-side code is written in C#, with JavaScript, HTML5, and CSS3 on the client-side. MD-CTS uses responsive design to deliver a consistent user experience across all mobile devices, tablets, and desktop computers.

To compare MD-CTS with paper-based medical dictionaries and the curated biomedical ontology of MeSH, we randomly selected 63 biomedical words from a collection of 50 articles of clinical and translational science published in the week of July 22, 2013. These articles were randomly selected from the following journals: *Science*, *Nature*, *Science Translational Medicine*, *New England Journal of Medicine*, and *Lancet*. Criteria for publication selection included occurrence of the publication date after latest construction of the MD-CTS lexicon from PubMed so those publications were not already indexed by MD-CTS. We reported the percentages of the randomly selected 63 biomedical words found in each of the paper-based medical dictionaries and in the curated biomedical ontology of MeSH as well as each of the four databases integrated into MD-CTS. We also reported the count of overlapped words in between Taber's, Stedman's and Dorland's paper-based dictionaries and the online MeSH resources with integrated access from MD-CTS.

## 3 Results

**Fig. 1:**
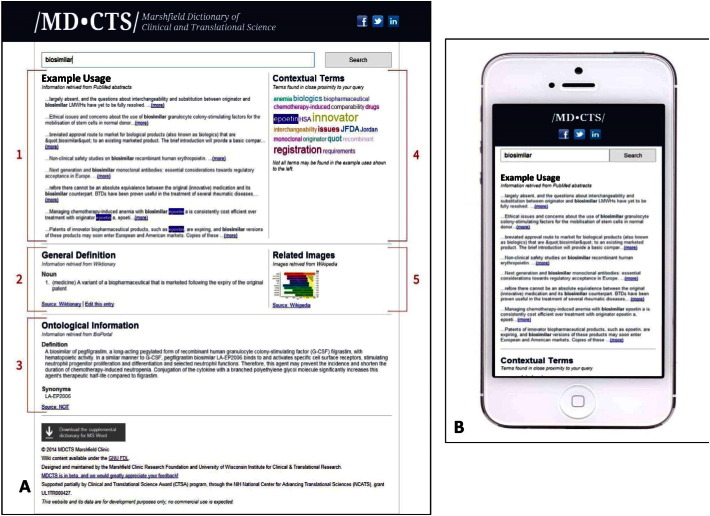
The design of the MD-CTS website. A) Desktop version on a computer screen. B) Mobile version on smaller screens such as SmartPhones.

We created MD-CTS, a mobile and desktop online reference, available for users across platforms and not constrained to a specific hardware or browser (Fig. 1). To help users search terminology and its usage, MD-CTS provides a simple query interface to display up to five different sections of information (Fig. 1A). The first section, named “Example Usage”, contains ten snippets from random Medline abstracts. These snippets assist the user in inferring the definition for the word from context. The second section, entitled “Definition,” includes a general definition of the word term that the MD-CTS retrieves from Wiktionary. The user also has an option to edit this entry or create a new entry in Wiktionary in the event that no definition is found. The third section, “Ontological Information,” illustrates the top results from interrogation of NCBO's BioPortal [4] (http://www.bioontology.org/), including ontological definition and synonyms for the targeted word. The fourth section, “Contextual Terms,” displays a tagcloud of alphabetized terms whose sizing appears in direct proportion to occurrence in the snippets returned from the “Example Usage” section. On mouse-over, each tagcloud term will be displayed to reflect its relative usage and its frequency in the “Example Usage” section. The fifth section is termed “Related Images.” The tool searches Wikipedia for any image that has a relationship with the targeted word. The responsive design of the website accommodates both desktop (larger screen) and mobile usage (smaller screen) displays using HTML5 (Fig. 1B).

We demonstrated the comprehensive coverage of MD-CTS using 63 randomly selected biomedical words in comparison with Taber's Cyclopedic Medical Dictionary (Taber), Stedman's Medical Dictionary (Stedman), Dorland's Illustrated Medical Dictionary (Dorland), and MeSH [2] (Table 1). The sizes of different sources (measured by number of entries) differ widely, ranging from 65,000 (Taber's) to 4,411,974 (Wiki). We found Wiktionary generally provided better coverage (73%) than paper-based dictionaries (57%–71%) which are updated less frequently. The MeSH ontology, maintained and updated regularly by the National Library of Medicine, demonstrated better coverage at 81%. Comparatively, BioPortal [4], a meta-search engine searching 372 biomedical ontologies (including MeSH), scored 84% coverage, while MD-CTS scored 95% on provision of usage examples among the 63 selected words. In Table 2, we have compared the words detected from paper-based dictionaries (Taber, Stedman, Dorland) and online MeSH ontology with the entity of the words available through MD-CTS which integrates four databases (see Table 1). The results show that all the words covered by Taber, Stedman, Dorland, and MeSH, are also covered by MD-CTS. MD-CTS covered 62 of the 63 words using all the combined databases (Wiktionary, Example Usage, Ontology, and relevant images).

## 4 Discussion

MD-CTS provides integrated access for clinical and translational scientists to look up emerging terms. The automated, computer-generated construct of MD-CTS enables near real-time update of emerging terms in the scientific literature but also imposes several limitations. First, computers lack intelligence to determine if a term falls within the scope of clinical and translational medicine. Second, for practicality, the source of new terms was limited to those identified in Medline abstracts, following exclusion of the top 5000 common English words. Third, MD-CTS stores and retrieves words at the root word level only. In a future version, phrases and hyphenated words can be considered for inclusion. Due to the labor-intensive nature of looking up words in paper-based dictionaries, we utilized a relatively small set of 63 words to evaluate the coverage of different reference sources. The difference in coverage may not be statistically significant, but the trend is informative.

In conclusion, we presented MD-CTS as an integrated reference tool for clinical and translational science. MD-CTS integrates several sources of information to provide users with concept definitions, ontological definitions, usage in context, related terms, and related images in one centralized resource. MD-CTS covers newly generated terms as they appear in the scientific literature. More importantly, a user can easily edit and update the definition of an emerging word using MD-CTS after reviewing the usage examples. Our current plan is to update MD-CTS quarterly. MD-CTS is accessible at http://spellchecker.mfldclin.edu/.

## Author contributions

JF, WR, CK, ZY and MRM contributed equally to this project; JF programmed the database; WR programmed the web and mobile interface; CK assisted in the web framework development and developed the statistical evaluation tool; MRM analyzed the results and drafted the manuscript; ZY designed the statistical evaluation; JB and YX contributed to the early pilot; DB, JS, and MP designed the look-and-feel; BF and BB evaluated the results and compared MD-CTS with traditional dictionaries; BW managed the project; CAS, UT, EM and SML conceived and designed the project; all authors wrote and reviewed the manuscript.

## Figures and Tables

**Table 1:** A comparison of frequently used medical dictionaries using the test list of 63 randomly selected biomedical words.

	Paper-based dictionaries			Online ontology	Integrated access from MD-CTS			
Data sources	Taber's Cyclopedic Medical Dictionary	Stedman's Medical Dictionary	Dorland's Illustrated Medical Dictionary	MeSH	Wiktionary	Example Usage	Ontology	Relevant Images
Number of entries	65,000	107,000	120,000	218,000 entry terms and 219,000 supplementary headings	523,157 entries	1,795,769 indexed terms	372 ontologies from BioPortal	4,411,974 articles from Wiki
Latest edition	22nd edition, 2013	28th edition, 2005	32nd edition, 2011	Annual update	Continuous crowdsourced update	2013, updated quarterly	Regular update at various intervals	Continuous crowdsourced update
% of the 63 medical words found	65%	57%	71%	81%	73%	95%	84%	22%

**Table 2:** A pairwise comparison of overlaps between dictionaries using 63 randomly selected biomedical words.

Dictionary 1	Dictionary 2	Number of shared word counts	Number of words in dictionary 1	Number of words in dictionary 2
Taber	Stedman	30	41	36
Taber	Dorland	37	41	46
Taber	MeSH	37	41	52
Taber	MD-CTS	41	41	62
Stedman	Dorland	36	36	46
Stedman	MeSH	33	36	52
Stedman	MD-CTS	36	36	62
Dorland	MeSH	42	46	52
Dorland	MD-CTS	46	46	62
MeSH	MD-CTS	52	52	62

Taber, Stedman, Dorland: paper-based dictionaries (Taber's Cyclopedic Medical Dictionary, Stedman's Medical Dictionary, Dorland's Illustrated Medical Dictionary).

MeSH: online ontology database.

MD-CTS: integrates data from Wiktionary, Example Usage (Medline), Ontology, Relevant Images (Wiki).

## References

[1] Zeng Q.T., Tse T. (2006). Exploring and developing consumer health vocabularies. J Am Med Inform Assoc.

[2] Resource N.C.B.I. (2014). Coordinators. Database resources of the National Center for Biotechnology Information. Nucleic Acids Res.

[3] Meyer C.M., Gurevych I., Granger S., Paquot M. (2012). Wiktionary: a new rival for expert-built lexicons? Exploring the possibilities of collaborative lexicography. Electronic Lexicography.

[4] Whetzel P.L., Noy N.F., Shah N.H., Alexander P.R., Nyulas C., Tudorache T. (2011). BioPortal: enhanced functionality via new Web services from the National Center for Biomedical Ontology to access and use ontologies in software applications. Nucleic Acids Res.

